# Effects of luteectomy in early pregnancy on the maintenance of gestation and plasma progesterone concentrations in the viviparous temperate lizard *Barisia imbricata imbricata*

**DOI:** 10.1186/1477-7827-8-19

**Published:** 2010-02-25

**Authors:** Martín Martínez-Torres, Marta E Hernández-Caballero, Juana Alba Luis-Díaz, Guadalupe Ortiz-López, Mario Cárdenas-León, Leticia Moreno-Fierros

**Affiliations:** 1 Laboratorio de Biología de la Reproducción-Unidad de Morfofisiología, Facultad de Estudios Superiores Iztacala, Universidad Nacional Autónoma de México, Los Reyes Iztacala, AP 314, Tlalnepantla Estado de México CP 54090, México; 2 Laboratorio de Biología de la Reproducción Humana, Hospital Juárez de México, Secretaría de Salubridad y Asistencia. Avenida Instituto Politécnico Nacional # 5160, Magdalena de las Salinas, AP 07760, México DF, México; 3 Laboratorio de Hormonas Proteicas, Departamento de Biología de la Reproducción, Instituto de Ciencias Médicas y de la Nutrición Salvador Subirán, México DF, México; 4 Laboratorio de Inmunidad en Mucosas-Unidad de Biomedicina, Facultad de Estudios Superiores Iztacala, Universidad Nacional Autónoma de México, Los Reyes Iztacala AP 314, Tlalnepantla Estado de México, CP 54090, México

## Abstract

**Background:**

Several studies have shown that the corpus luteum is the principal source of progesterone during the gravidity period in reptiles; however, its participation in the maintenance of gestation in the viviparous squamata is in dispute. The effects of ovariectomy or luteectomy vary according to the species and the time at which the procedure is performed. In this paper, we describe the effects of luteectomy during early pregnancy on the maintenance of gestation and progesterone concentrations in the temperate Mexican viviparous lizard *Barisia imbricata imbricata.*

**Methods:**

Twenty-four lizards were subjected to three different treatments: luteectomy, sham luteectomy or non-surgical treatment, and blood samples were obtained before and after surgical treatment at different stages of gestation to determine the effects of luteectomy on the maintenance of gestation and progesterone concentrations.

**Results:**

Spontaneous abortion was not observed in any of the females. However, luteectomy provoked abnormal parturition and a significant reduction in the number of young born alive. Parturition was normal in untreated females as well as those submitted to sham luteectomy. The surgical treatment also caused a significant reduction in progesterone concentrations in luteectomised females during early and middle gestation. However, no significant differences in hormone concentrations were observed among the three groups during late gestation or immediately post-parturition.

**Conclusions:**

Our observations indicate that the presence of the corpus luteum is not necesary for the maintenance of gestation, but that it does participate in parturition control. Moreover, the corpus luteum of the viviparous lizard *B. i. imbricata* produces progesterone, at least during the first half of pregnancy, and that an extra-ovarian source of progesterone must maintain gestation in the absence of luteal tissue.

## Background

The corpus luteum (CL) is an ephemeral endocrine gland that is present in gravid vertebrate females [1]. Several authors agree that embryo retention in gravid reptiles is controlled by the activity of the CL through its capacity to produce progesterone (P_4_) [1]. In oviparous reptiles, there is a positive correlation between luteal function and the duration of egg retention [2], as luteal regression occurs just before (*Pseudemys scripta *[3]; *Chelydra serpentina *[4]; *Sceloporus anneus *[5]) or immediately after oviposition (*Calotes versicolor *[6]; *Uromastick hardwicki *[7]; *Naja naja *[8]). Furthermore, surgical removal of the luteal tissue can cause premature oviposition and a reduction in P_4 _concentrations (*Sceloporus undulatus*, [9]; *Cnemidophorus uniparens *[10]). However, viviparity requires that the period of the egg retention *in utero *be extended for the entirety of embryonic development. In viviparous squamata species, no direct correlation exists between luteolysis and parturition, since luteal regression can occur at any time during pregnancy and differs among species [1,2]. Removal of the ovaries or the CL in gravid viviparous lizards has different effects depending on the species and stage of pregnancy when the treatment is applied. Luteectomy and ovariectomy both induced spontaneous abortion when performed during early pregnancy in *Xantusia vigilis *[11] and *Sceloporus jarrovi *[12]. However, the same treatment in *Lacerta vivipara *[13] and *Sceloporus cyanogenys *[14,15] did not provoke abortion, but caused abnormal parturition (stillborn young and delayed parturition). In other species, such as *Mabuya carinata *[16] and *Chalcides ocellatus *[17], neither the extirpation of luteal tissue nor ovariectomy affected the length of gestation or parturition. These results indicate that it is unclear whether the CL participates in the maintenance of gestation in viviparous lizards. *Barisia imbricata imbricata *is a viviparous temperate lizard endemic to México. For this species, vitellogenesis begins in late summer (late August or early September), mating takes place in October and ovulation occurs during November and early December [18]. Gestation lasts throughout the winter and a substantial part of the spring [19,20]. Luteal tissue develops after ovulation during the first third of gestation and persists throughout the pregnancy [20], though the first regressive changes are observed early in the second third. The regression of luteal tissue advances gradually, and parturition does not occur until late May or June [21]. However, several evidences suggest that the CL is the main site of P_4 _production during gestation in *B. i. imbricata *[20]. The activity of 3β-hydroxyl steroid dehydrogenase Δ^5-4 ^isomerase (Δ^5-4 ^3β-HSD) has been detected in the CL of *B. i. imbricata *throughout gestation. A positive correlation has also been observed among the histological appearance of luteal tissue, the histochemical activity of Δ^5-4 ^3β-HSD and P_4 _plasma concentrations during gestation [20]. These data suggest that in *B. i. imbricata*, the CL is the principal P_4 _source during pregnancy, but its role in the maintenance of gestation remains unknown. The goal of this study was therefore to determine the effects of luteectomy during early pregnancy on the maintenance of gestation and P_4 _concentrations in the viviparous temperate lizard *B. i. imbricata*.

## Methods

### General

Twenty-four adult females (9.5 - 13.5 cm in snout-vent length, 28.9 ± 5.8 g) of *B. i. imbricata *were collected from Cuautitlán, State of Mexico (19° 37'' N; 99° 11'' W; 2253 m altitude) during the first half of December 2003. All experimental procedures were approved by the Bioethical Committee of the FES Iztacala UNAM. The lizards were toe-clipped for individual identification and transported to the laboratory on the same day as collection. The following week, ovulation was determined by ultrasonography using a linear ultrasound unit with variable capacity from 5-10 MHz [18]. Only females exhibiting oviductal eggs were utilised. These females were randomly assigned to three groups: (1) luteectomy (n = 9), (2) sham luteectomy (n = 7) and (3) intact females (n = 8).

### Lutectomy effect on the maintenance of pregnancy

The week after ultrasound scanning, the lizards in the luteectomy and sham luteectomy groups were anaesthetised with ether and a ventrolateral incision was performed. In the females submitted to luteectomy, the ovaries were exposed, all CL were surgically removed from each ovary and the total number of CL was registered. One CL of each female was fixed in 10% buffered formol and processed for routine histology. Lizards submitted to sham luteectomy were treated identically, but the CL were not removed and only the numbers of CL were registered. When the surgical treatments were completed, the lizards were sutured and each female was deposited in an individual terrarium in our laboratory with free access to water and food (mealworms of *Tenebrio*, wax worms of *Galleria mellonela*, domestic crickets of *Achaeta *spp. and grasshoppers) for three days following surgery to allow for their recovery. On the day of the surgery, each lizard received an injection of 5000 U. I. of penicillin G and on the following two days, they received 500 μg streptomycin sulfate (I. M.). The group of intact lizards did not receive surgical treatment, but were given the antibiotics. After the recuperation period, all females were housed in individual terrariums (30 × 50 × 30 cm) and kept throughout the pregnancy period in the greenhouse of the UMF-FES Iztacala UNAM (19° 36' N, 98.5° 11' W and 2240 altitude). The lizards had unrestricted access to water and food and were maintained at a temperature and natural photoperiod throughout the experiment. The terrariums were scrutinised daily from the day of surgery until the time of parturition to detect early embryo expulsion or non-viable eggs (see definitions below). The dates of abortions, expulsion of non-viable eggs and parturition as well as the number of birth products (live young, stillborn embryos or nonviable eggs) were registered. We also recorded whether parturition was normal or abnormal. Each lizard was dissected three weeks after the birth of the young. In luteectomised females, we verified that the CL was missing in the ovary and that the uterus was devoid of embryos or nonviable eggs. In the sham-luteectomised lizards, the uteri were reviewed in the same way. In intact lizards, the number of CL was recorded in addition to the state of the uterus. Finally, because oophagy has been reported to occur in this species [22], the stomachs of all lizards were also examined to determine whether the females ate the embryos or nonviable eggs.

We defined criteria to assess the effects of deluteinisation on the maintenance of gestation in *B. i. imbricata *according to those proposed by Panigel [13] for *Lacerta vivipara *and Callard et al [15]. for *Sceloporus cyanogenys*: **Abortion **was diagnosed if A) the embryo or foetus (alive or dead) was expulsed after surgical treatment (luteectomy or sham luteectomy) but before reaching stage 40 of development or B) if the expulsion of the embryo or foetus occurred in intact females (maintained in the same environmental conditions as the females submitted to surgery) during any period of development before stage 40. **Normal parturition **was diagnosed if the offspring had completed embryonic development (e.g., reached stage 40) and were expulsed alive with the physiological capacity for life within 48 h from the first expulsion of young. **Abnormal parturition **was diagnosed if one of several scenarios were observed: A) premature parturition: if the young was expulsed after reaching stage 40 of development, but at the time of expulsion, the foetus presented yolk sac residue (premature young) B) dissociate parturition: if all young completed embryonic development but were expulsed over a period of more than 48 hours; C) delayed parturition: if the young completed embryonic development but were expulsed dead; or D) if at the surgical inspection or autopsy performed 21 days after the first young was born embryos (alive or dead) were found within the uterus.

### Corpus luteum histology

Fixed corpora lutea were washed in running water, dehydrated in an alcohol gradient, cleared in xylene and embedded in paraplast. Histological sections (7 μm) were cut and stained using Harri's haematoxylin and eosin [23]. The sections were examined by microscopy to determine the stage of development according to Martínez-Torres et al., [20].

### Effects of luteectomy on progesterone concentrations

About 2-3 hours before surgery (luteectomy or sham luteectomy), a blood sample of 200 ± 10 μl was obtained from each female by intracardiac puncture with a heparinised syringe [20]. Blood samples were also obtained at 24 hours as well as 8 (early gestation), 16 (middle gestation) and 24 weeks (late gestation) after surgical treatment (luteectomy or sham luteectomy) and one day after parturition (immediate postpartum). Intact females were bled to the same volumes and at the same times as lizards in other treatment groups. The blood was centrifuged immediately after collection, and the plasma was decanted and frozen at -40°C until the P_4 _radioimmunoassay was performed, according to the methods of Martínez-Torres et al. [20]. All aliquots were obtained between 9.00 and 12.00 hours.

### Radioimmunoassay

Plasma progesterone concentrations were quantified using a commercial kit (Coat-A-Count Progesterone, Diagnostic Products Corporation, Ca 90045). The assay was performed in duplicate using 50 μl of plasma without prior dilution or extraction. ^125^I-labelled progesterone was supplied as the reactive tracer. The antiserum was specific for progesterone. Steroids showing cross-reactivity (relative to progesterone, 100%) were androstenediol (non-detectable), corticosterone (0.9%), cortisol (0.03%), 11-deoxicorticoesterone (2.2%), 20α-dihydroprogesterone (0.2%), estradiol (nondetectable), 17α-hydroxyprogesterone (3.4%), 5β-pregnan-3α-ol-20-one (0.05%), 5α-pregnan-3, 20-dione (3.2%), pregnenolone (0.1%), and testosterone (0.1%). Inter- and intra-assay coefficients of variation were 7.9 and 7.6%, respectively. The sensitivity of the assay was 0.02 ng/ml. The values were obtained using GAMBYT software.

### Statistics

To determine differences in litter size and number of offspring born alive as well as to compare the number of CL among the treatment groups, we used a one-way analysis of variance (ANOVA). A two-way ANOVA for repeated measures was used to determine significant changes in P_4 _concentrations. Post hoc tests were carried out using the Tukey method to determine differences; the significance level was set at p < 0.05 [24]. All statistical analyses were performed with SigmaStat software (version 3.5 for Windows).

## Results

Corpora lutea obtained from luteectomised lizards showed, upon histological examination, according to Martínez-Torres et al. [20], histologic characteristics of active immature glands, except for two CL that resembled mature glands. These results agree with Martínez-Torres et al. [20]. Microscopic analyses also showed that the CL were in several stages of luteal development. The CL of one lizard were in stage I, whereas the CL of four lizards were in stage II, two were in stage III, and two were in stage IV. Corpora lutea were constituted by the luteal cell mass surrounded by the theca interna and the theca externa. In the luteal cell mass, we observed some cells with light nuclei, with one, two or three nucleoli and acidophilic cytoplasm as well as others with condensed chromatin. These characteristics, according to Martínez-Torres et al. [20] and Saidapur [25], indicate that the luteal tissues were actively synthesizing steroids. A cavity was present in the luteal cell mass in all extirpated CL, except in the CL that were in stage IV. No CL showed any degenerative characteristics (luteolysis). We did not observe significant differences in CL number among the three treatment groups [luteectomy: 14.1 ± 1.6, sham luteectomy: 15.5 ± 2.6, intact lizards: 14.5 ± 2.3; F(2, 21) = 8.73, p > 0.41].

### Effects of luteectomy on the maintenance of gestation

We observed no abortion in any females; however, all luteectomised females showed abnormal parturition (Table 1). Significant differences in clutch size were not observed among luteectomised, sham-luteectomised and intact lizards [luteectomy: 11.8 ± 2.7, sham-luteectomy: 14.1 ± 3.0, intact: 13.8 ± 2.5; F(2, 21) = 1.63, p > 0.219, Table 1], although a statistically significant reduction was observed in the number of young born alive from luteectomised lizards [luteectomy: 9.8 ± 2.3, sham-luteectomy: 13.1 ± 2.6, intact: 13.6 ± 2.4; F(2, 21) = 1.63, p < 0.05].

**Table 1:** Effects of luteectomy on several parameters of the gestation period of the viviparous lizard *Barisia imbricata imbricata*

Treatment	Abnormal parturition	Mean of corpora lutea	Mean of birth products total number	Mean of viable young born	Mean of stillborn and premature young	Mean of non-viable eggs
Luteectomy (n = 9)	9	14.1 ± 1.6 (n = 127)	11.8 ± 2.7 (n = 107 100%)	9.8 ± 2.3^**a **^(n = 91 85.04%)	0.8 ± 0.3 (n = 10 9.34%)	1.2 ± 1.4 n = 6 5.6%
sham luteectomy (n = 7)	2	15.5 ± 2.6 (n = 108)	14.1 ± 3.0 (n = 99 100%)	13.1 ± 2.6^**b **^(n = 92 92.9%)	0.3 ± 0.4 (n = 3 3.03%)	0.6 ± 1.2 (n = 4 4.04%)
None (n = 8)	1	14.5 ± 2.3 (n = 116)	13.8 ± 2.5 (n = 111 100%)	13.6 ± 2.4^**c **^(n = 109 98.1%)	0.09 ± 0.03 (n = 1 0.9%)	0.09 ± 0.03 (n = 1 0.9%)

^**a **^is significantly minor with respect to ^**b **^and ^**c **^at p < 0.05

Delivery of offspring from luteectomised females occurred 26-30 weeks after surgical treatment and lasted for 5-19 days for each female. The newborns were fully developed (stage 40 according to Deffaure and Hubert [26]). However, some stillborn and live-born young were surrounded by extraembryonic membranes and yolk sacs containing vitelus (Table 1). All premature young that were born alive died the same day. We also observed that luteectomised females expulsed non-viable eggs during parturition. Parturition in the sham-luteectomised lizards initiated 27 weeks after surgery in three lizards, 28 weeks in two lizards and 30 weeks in two lizards. Five sham-luteectomised lizards showed normal parturition while the remaining females exhibited abnormal parturition. Normal parturition was observed in all but one intact pregnant lizard. Parturition in the intact lizards occurred 27 weeks after the other lizards were subjected to surgery in two lizards, 29 weeks in three lizards, 30 weeks in one lizard and 31 weeks in two lizards. When parturition was normal in the intact and the sham-luteectomised lizards, all offspring were expulsed in just one day.

All females were submitted to laparotomy 21 days after the expulsion of the first offspring in order to examine the ovary, uterus and stomach. Only one luteectomised female had retained young in her uterus. We observed that one deluteinised lizard ate one premature embryo and another ate one nonviable egg. The uteri of sham luteectomised and intact lizards were devoid of embryos. However, we detected a nonviable egg in the stomach of one sham-luteectomised lizard and one fully developed embryo in another sham luteectomised lizard. Finally, there was a nonviable egg in the stomach of one intact female, and one young stillborn was observed in another.

### Effects of luteectomy on progesterone concentrations

**Figure 1:**
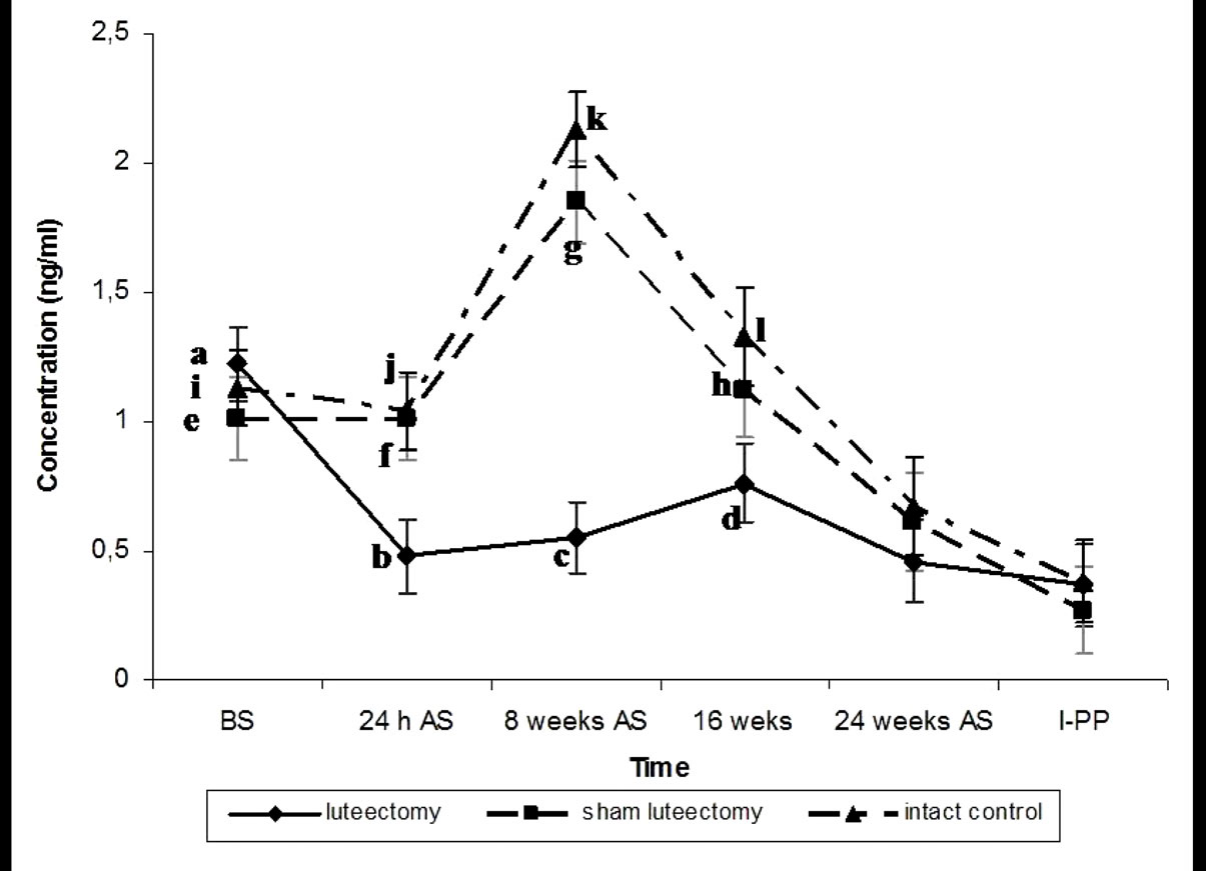
**Effect of luteectomy on the progesterone plasma concentration in viviparous lizard Barisia imbricate**. Patterns of progesterone plasma concentrations in luteectomised, sham luteectomised and intact pregnant females of the viviparous lizard *Barisia imbricata imbricata*. BS: before surgery, W-AS: weeks after surgery, I-PP: immediately post-parturition ^**a **^is significantly higher with respect to remaining times of gestation of luteectomised lizards group at p < 0.01; ^**g **^is significantly higher with respect to remaining times of gestation of sham luteectomised lizards group at p < 0.01; ^**k **^is significantly higher with respect to remaining times of gestation of intact lizard group at p < 0.01; ^**b **^is significantly less with respect to ^**f **^and ^**j **^at p < 0.01; ^**c **^is significantly less with respect to ^**g **^and ^**k **^at p < 0.01; ^**d **^is significantly less with respect to ^**l **^at p < 0.01.

The patterns of P_4 _concentrations in luteectomised, sham-luteectomised and intact females are shown in Figure 1. We found that luteectomy provoked a significant reduction in plasma P_4 _concentrations when values in the early and middle stages of gestation were compared in sham-luteectomised [F(2, 5) = 15.2, p < 0.001] and intact lizards [F(2, 5) = 19.1, p < 0.001] (Fig. 1, Table 2). We did not observe any significant differences between the intact control and the sham-luteectomised lizards throughout the stages of gestation [F(2, 5) = 4.41, p = 0.38] (Fig. 1, Table 2).

**Table 2:** Progesterone plasma concentrations^1 ^in luteectomised, sham luteectomised and intact pregnant females of viviparous lizard *Barisia imbricata imbricata*

Treatment	before surgery	24 h after surgery	8 weeks after surgery	16 weeks after surgery	24 weeks aftersurgery	immediate postpartum
luteectomy	1.22 ± 0.14 (n = 9)	0.48 ± 0.15^**f **^(n = 9)	0.55 ± 0.14^**g **^(n = 8)	0.76 ± 0.15^**h **^(n = 9)	0.46 ± 0.16 (n = 7)	0.47 ± 0.16 (n = 7)
sham luteectomy	1.01 ± 0.16 (n = 7)	1.01 ± 0.16^**d **^(n = 7)	1.85 ± 0.16**^e ^(n = 7)	1.12 ± 0.18 (n = 6)	0.64 ± 0.19 (n = 5)	0.27 ± 0.17 (n = 7)
Intact control	1.13 ± 0.15 (n = 8)	1.04 ± 0.15^**a **^(n = 8)	2.13 ± 0.15*^b ^(n = 8)	1.33 ± 0.16^**c **^(n = 7)	0.67 ± 0.19 (n = 5)	0.38 ± 0.16 (n = 8)

^**1 **^values are in ng/ml.

**n **= number of blood samples used.

*^, ^** = Peak of P_4 _levels; the means was significantly higher at p < 0.01 with respect to remaining times of pregnancy examined (whiting to the same group).

^**f **^is significantly less with respect to ^**a **^and ^**d **^at p < 0.01.

^**g **^is significantly less with respect to ^**b **^and ^**e **^at p < 0.01.

^**h **^is significantly less with respect to ^**c **^at p < 0.01.

Progesterone concentrations prior to surgery were similar in all three groups [luteectomised: 1.22 ± 0.14 ng/ml, sham-luteectomised 1.01 (0.16 ng/ml, intact females 1.13 (0.15 ng/ml] (Table 2). In luteectomised lizards, P_4 _concentrations were significantly diminished within 24 hours following surgery. However, in sham-luteectomised and intact control lizards, P_4 _concentrations were similar to those recorded before surgery and significantly higher than the corresponding values from the luteectomised females [luteectomy: 0.48 ± 0.15 ng/ml, sham-luteectomy: 1.01 ± 0.16 ng/ml, intact lizards 1.04 ± 0.15 ng/ml; luteectomy vs. sham luteectomy: p < 0.047, luteectomy vs. control intact p < 0.031] (Fig. 1, Table 2). In luteectomised lizards, low concentrations of P4 were maintained at eight weeks after surgery (0.55 ± 0.14 ng/ml) and were not significantly different than the values recorded 24 hours after surgery (p > 0.73, Fig. 1). Conversely, in the sham-luteectomised and the intact control lizards, P_4 _concentrations significantly increased and reached the highest concentrations typically observed during gestation [sham-luteectomised 1.85 ± 0.16 ng/ml, intact lizard: 2.13 ± 0.15 ng/ml]. These values were significantly different than those detected in luteectomised lizards at the same time point [luteectomy vs. sham luteectomy: p < 0.001, luteectomy vs. intact control: p < 0.001]. However, at mid-gestation, P_4 _concentrations had diminished in the sham-luteectomised and intact lizards. In the luteectomised lizards, the P_4 _concentrations 16 weeks post-luteectomy were also low [0.76 ± 0.15 ng/ml], and we determined that they were not significantly different from those of sham-luteectomised females [1.12 ± 0.18, p = 0.2], but were indeed different than the concentrations observed in intact lizards [1.33 ± 0.16 ng/ml, p = 0.037]. In late gestation and immediately post-parturition, P_4 _concentrations were further diminished in all lizards, and significant differences were not observed among the three groups (Fig. 1, Table 2).

## Discussion

Several researchers have suggested that the CL plays a central role in maintaining the gestation and the evolution of reptilian viviparity, given its capacity for P_4 _production [27-31]. Moreover, several authors have reported evidence suggesting that this gland is the major source of P_4 _during pregnancy in many species of viviparous lizards and snakes [1,20,32,33]. However, a number of experimental studies in several species of viviparous squamata have shown that while the deluteinisation causes significantly diminished P_4 _concentrations, in no species thus far investigated does this treatment completely eliminate P_4 _in the plasm [32,34]. In addition, the deluteinisation produces varying responses depending on the species and time of gestation at which the surgery is performed [12,30]. We removed the CL during early pregnancy of *B. i. imbricata *and we observed a significant reduction in P_4 _after luteectomy, but hormone concentrations never fell below the limit of detection. Moreover, we observed no abortions; however, all luteectomised lizards showed abnormal parturition. We also observed that histological examination of the extirpated luteal tissues exhibited active endocrine glands midway through the maturation process, in accordance with results from Martínez-Torres et al. [20] and Saidapur [25]. These observations indirectly show that the CL continues to participate in the production of P_4 _in *B. i. imbricata*. We think that these results also suggest that the primary importance of this gland has been overshadowed due to the emergence of extraluteal structures (i.e., glandula adrenal), during the evolution of viviparity, capable of producing this steroid and to maintain gestation.

Luteectomy in the lizard *Tiliqua rugosa *[31,32] and in the garter snake *Thamnophis elegans *[33,34] produced results similar to those observed in our study. Such observations led at several researchers [31-34] at the same conclusion regarding an extra-ovarian supply of P_4_, and they suggest the adrenal gland (AG) as a candidate source. Highfill and Mead [33] found that adrenalectomy reduces P_4 _concentrations to a non-detectable state in non-pregnant snakes. Likewise, in *Lacerta vivipara*, Dauphin-Villemant and Xavier [35] and Dauphin-Villemant et al., [36] observed *in vitro *and *in vivo *an increase in the adrenal activity and production of P_4 _during gestation. These observations support the idea that the AG may participate in the maintenance of gestation in viviparous lizards. This may hold true for *B. i. imbricata*, though no studies have yet examined AG activity during gestation.

Other authors have suggested that atretic vitellogenic follicles (AVFs) may be an important ovarian source of P_4 _[37,38]. Villagran-Santa Cruz [37] observed an increase in the number and volume of AVFs in the second half of gestation in *S. mucronatus*, coinciding with the degeneration of the CL. Moreover, Guillette et al. [38] showed a positive correlation between plasma P_4 _concentrations and AVFs number as well as the presence of placenta chorioallantois (PCA) in the second part of gestation in *S. jarrovi*. In our study, we observed that three females submitted to luteectomy and two females submitted to sham-luteectomy did not contain any AVFs. This observation rejects the possibility that AVFs participate in the maintenance of gestation in this lizard species. Several studies have shown that the PCA of some lizard species (*Chalcides chalcides*, [39]; *Sceloporus jarrovi*, [40]) have endocrine capacity. Guarino et al. [39] and Painter and Moore [40] observed that the PCA is an important source of P_4 _in late pregnancy and that it coincides with CL regression in these species. It is unknown whether the PCA of *B. i. imbricata *embryos have the capacity for P_4 _production. However, Martínez-Torres et al. [21] recently found that the omphaloplacental residue of new born of *B. i. imbricata*, stained positive for Δ^5-4 ^3β-HSD activity. This observation suggests that this structure is capable of metabolising or producing P_4_. However, we do not know the stage of gestation and/or embryonic development when omphaloplacenta acquire this ability. Similarly, we do not know which embryonic stage corresponds to the time of deluteinisation. We expected the AG to be the secondary source of P_4 _in *B. i. imbricata *(as claimed by other researchers for other lizard species), at least during early gestation, because we observed that the embryos of the lizards whose CL were in initial stages I and II were in the cicatricela stage (segmentation or gastrulation, according to Defaure and Hubert [26]). Therefore, these young embryos have neither a placenta nor extra-embryonic membranes. However, we do not discard the possibility that the placenta may participate in the production or the metabolism of this hormone at some time during gestation.

Parturition has been widely studied in several mammalian species (for review, see [41,42]); however, few papers have addressed this process in reptiles (for review, see [13,34,12,43,44]). Nevertheless, several authors agree that the stimulation of uterine contraction associated with parturition involves a complex interaction between several hormones steroids (P_4 _and estradiol 17β), arginine-vasotocine (AVT) and prostaglandins [45,46]. Further evidence suggests that the CL may influence parturition in squamata since luteectomy provokes abnormal parturition in some species (*Lacerta vivipara*, [13], *S. cyanogenys *[15], *T. sirtalis*, [32]). In *B. i. imbricata *we found a similar situation. The expulsion of premature, stillborn young and dissociated parturition by luteectomised alligator lizards suggests that hormones from the CL (P_4_, estradiol 17β or other hormones) participate in the control of parturition. In the absence of CL, the uterine contraction may be perturbed and therefore, the delivery of all pupetts occurred over a prolonged period of time (5-19 day). Moreover, these results show the importance of the CL in the modulation of contractile activity of the myometrium of *B. i. imbricata*.

According to Jones and Guillette [2], luteal steroids modulate the response of oviductal muscles to AVT, especially in late pregnancy. Moreover, Ferguson and Bradshaw [32] observed that the circulating plasma concentrations of AVT increase in pregnant *Tiliqua rugosa *lizards as the time of parturition approaches and that plasma P_4 _concentrations decline and CL degeneration proceed concomitant with this AVT elevation. In a previous paper, Martínez-Torres et al. [20] showed that in *B. i. imbricata*, plasma P_4 _concentrations are higher in early pregnancy and then drop gradually throughout gestation and that there is a positive correlation between P_4 _concentrations and the histological activity of the CL. In this study, we observed a similar situation in sham-luteectomised and intact pregnant lizards. In contrast, we saw a significant reduction in P_4 _concentrations after surgery in luteectomised lizards, and these low levels were maintained for the rest of gestation. However, we found that the P_4 _values during late gestation of luteectomised lizards were not significantly different than the concentrations determined in sham-luteectomised and intact lizards. These data suggest two conclusions: A) that the quiescent uterus necessary for embryo retention may be maintained by low levels of P_4 _and B) that the reduction of P_4 _in the first half of gestation and/or the absence of other hormones from the CL (e.g., estradiol 17β) might alter uterine sensitivity to neurohypophyseal hormones (e.g., AVT), which consequently might cause abnormal parturition. Further studies are needed to determine how other progesterone, luteal hormone and arginine vasotocine levels fluctuate throughout gestation, in order to define the role of the CL in the maintenance of gestation.

Guillette and Casas-Andrew [19] notified that gestation in *B. i. imbricata *lasts for approximately six months. However, in our study, we observed that gestation could last for as long as seven months, since parturition occurred in all females between 27 and 31 weeks after that the presence of oviductal eggs was confirmed by ultrasound scanning. Precise determination of the duration of gestation is very important, as it would inform the study of the hormonal changes associated with the maintenance of gestation as well as the mechanism that controls parturition.

## Conclusions

In accordance with all observations described above, we arrived at the following conclusions: A) the CL of *B. i. imbricata *is capable of P_4 _production, B) a secondary extra-ovarian source of P_4 _capable of maintaining gestation must exist, C) the CL participates in the modulation of parturition, and D) given the data obtained in ur study and that reported by other authors, we presume that the level of CL participation in the maintenance of gestation in viviparous lizards differs across species.

## Competing interests

The authors declare that they have no competing interests.

## Authors' contributions

MMT and LMF conceived, designed and coordinated the study. MMT moreover wrote the manuscript and completed the statistical analysis. Moreover, LMF also supervised the radioimmunoassay, and revised the manuscript. MMT and MEHC collected all lizards and blood samples, and performed surgical procedures. JALD carried out all tissue processing, histological analysis and ultrasound scanning and participated in the analysis and discussion of the results. GOL and MCL carried out the radioimmunoassays and critical revisions of the manuscript. All co-authors provided inputs during final manuscript preparation. All authors read and approved the final manuscript.
